# Environmental investigation of a hepatitis A outbreak associated with spring water consumption in an Anabaptist community: Kentucky, 2018–2019

**DOI:** 10.2166/wh.2026.248

**Published:** 2026-01-30

**Authors:** Shanna Miko, Amy Kahler, Alexis Roundtree, Katharine Benedict, Monique Foster, Douglas Thoroughman, Amanda Odegard, Jennifer Khoury, Carrell Rush, Mohammad Zafrullah, Tonya Hayden, Michael Purdy, Lilia Ganova-Raeva, David Williams, Layne Mounsey, Adam Massey, Mia Mattioli

**Affiliations:** a Division of Foodborne, Waterborne, and Environmental Diseases, Centers for Disease Control and Prevention, Atlanta, GA, USA; b Division of Viral Hepatitis, Centers for Disease Control and Prevention, Atlanta, GA, USA; c Division of State and Local Readiness, Centers for Disease Control and Prevention, Atlanta, GA, USA; d Kentucky Department for Public Health, Frankfort, KY, USA; e Allen County Health Department, Scottsville, KY, USA

**Keywords:** coliphage, crAssphage, drinking water, groundwater, hepatitis A virus, septic system, spring water, wastewater

## Abstract

In 2019, a hepatitis A outbreak in an Anabaptist community was reported to the Kentucky Department for Public Health. Epidemiological data suggested untreated spring water as a potential outbreak exposure. An environmental investigation was conducted to evaluate this less common hepatitis A virus (HAV) exposure source. We collected water samples from the main spring used for drinking and produce irrigation by the primary household where cases were identified, from springs feeding the main spring, and from the household’s gray water discharge and human waste systems. Samples were tested for HAV, and viral and bacterial fecal indicators. HAV was detected in the main spring water and downstream of the household gray water discharge pipe. HAV was repeatedly detected in the follow-up samples from spring water and in wastewater from a new septic system. The environmental investigation confirmed HAV contamination of the household drinking water. Human-specific and general fecal indicators indicated a hydrological connection between the waste system and the drinking water source. Given that private springs are unregulated in the United States, spring water should be considered as a risk factor in HAV and other waterborne virus outbreaks within communities less likely to utilize water treatment systems.

## GRAPHICAL ABSTRACT

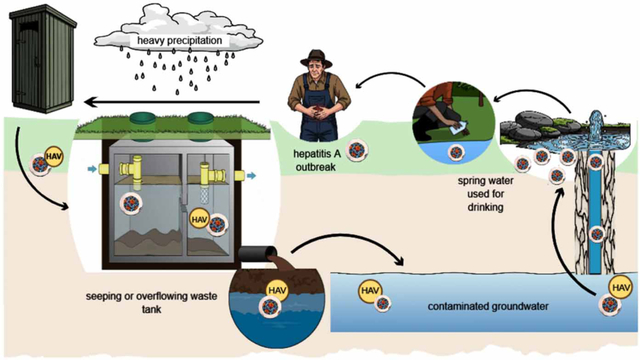

## INTRODUCTION

Private springs are the sole source of drinking water and water for other household needs for some homes in rural areas (Johnson *et al*. 2019). However, the quality and safety of water from privately owned sources are not regulated under federal or, in most instances, state laws, and therefore it may not be routinely tested or disinfected (U.S. Geological Survey 2019; Lawton 2024; Schmitt *et al*. 2024). Persons drinking untested and untreated groundwater may be exposed to elevated concentrations of some contaminants, including infectious pathogens (DeSimone 2009; Lee & Murphy 2020; Mattioli *et al*. 2021). A U.S. government survey reported that .20% of 2,100 private wells tested contained one or more biological or chemical contaminants at levels greater than human-health benchmarks (DeSimone 2009). Untreated groundwater sources were associated with a third of drinking water outbreaks reported to the U.S. Centers for Disease Control and Prevention (CDC) during 1971–2008 (Wallender *et al*. 2014). Further, there were 32 reported hepatitis A outbreaks during 1971–2017 associated with drinking water from springs or wells (Barrett *et al*. 2019).

Hepatitis A is a vaccine-preventable, acute liver disease caused by the hepatitis A virus (HAV) (Nelson *et al*. 2020; Castaneda *et al*. 2021; Migueres *et al*. 2021). Symptoms of hepatitis A include fever, jaundice, fatigue, nausea, vomiting, stomach pain, joint or muscle pain, diarrhea, and anorexia (Nelson *et al*. 2020). Severity of disease increases in persons who are older, have weakened immune systems, chronic liver disease, or other health conditions (Collier *et al*. 2015; Nelson *et al*. 2020). Post-exposure prophylaxis within 2 weeks of exposure may prevent disease in previously unvaccinated persons aged .12 months (Nelson *et al*. 2020; Migueres *et al*. 2021); treatment for acute HAV infection is limited to managing symptoms with supportive care. HAV is an RNA virus typically spread via the fecal–oral route (Nelson *et al*. 2020; Castaneda *et al*. 2021; Migueres *et al*. 2021).

In high-income countries, person-to-person spread is often associated with outbreaks, and foodborneand waterborne-associated outbreaks are sporadic (Migueres *et al*. 2021). In the United States, drinking water-associated hepatitis A outbreaks have decreased with universal childhood vaccination and public health drinking water regulations (Barrett *et al*. 2019). Those at increased risk of acquiring HAV infection in the United States include men who have sex with men, persons who report drug use, persons experiencing homelessness, unvaccinated persons, and, in rare instances, those who drink untreated water (Barrett *et al*. 2019; Foster *et al*. 2019). In 2016, the United States began experiencing an unprecedented person-to-person hepatitis A transmission that caused a national outbreak lasting years, principally among men who have sex with men, the homeless, and the incarcerated (Hofmeister *et al*. 2021).

**Figure 1 |:**
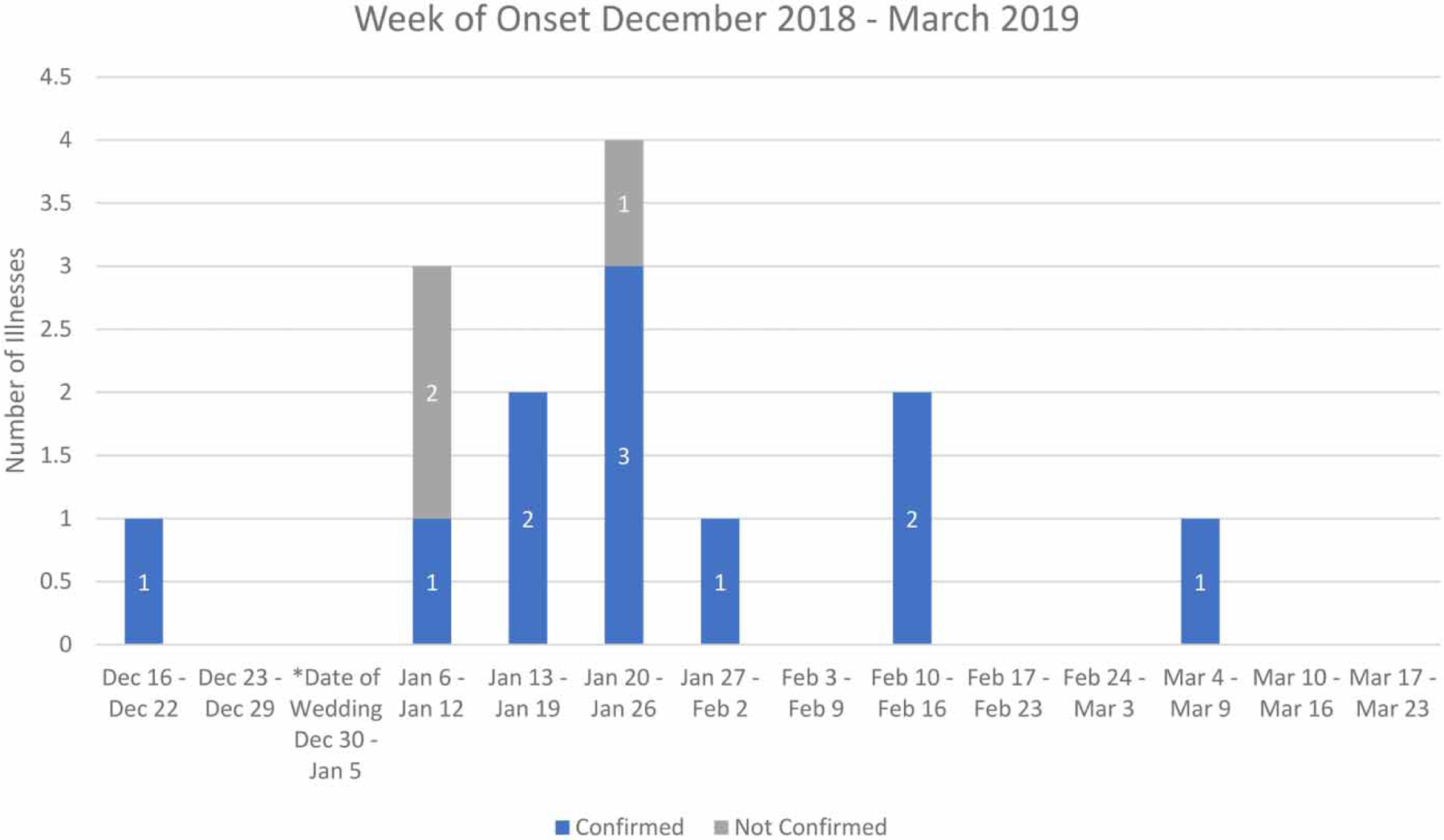
Epidemiological curve of hepatitis A illnesses by week of onset, Kentucky.

In February of 2019, the Allen County Health Department (ACHD) and Kentucky Department for Public Health (KY DPH) contacted the CDC for environmental support in their investigation of a hepatitis A outbreak among unvaccinated Anabaptist community members in Allen County, Kentucky, and neighboring Tennessee. Vaccination is not a widely accepted practice in some Anabaptist communities (Garrett-Wright *et al*. 2016; Anderson & Potts 2020; Anderson *et al*. 2023). Twenty-two people with HAV infections from two states were included in the outbreak, including 14 in Allen County (Figure 1). Among the persons with hepatitis A in Allen County, 11 were laboratory-confirmed. The ACHD and KY DPH conducted an epidemiological investigation and interviewed those who were ill to identify common exposures preceding illness onset.

Five of the hepatitis A outbreak-associated cases occurred in members of a seven-person family living in a single household. The primary source of water for the household was a natural spring. Some Anabaptist communities reject ‘modern’ technology and live in rural settings, and as a result do not utilize modern sewage systems or municipal drinking water (Anderson 2013; Amraotkar *et al*. 2015). Epidemiologic data, collected by ACHD, suggested water consumption from the home’s spring as a potential source of exposure associated with the outbreak. Everyone in the home drank the spring water except an adult man with an unstated medical condition, who did not report symptoms of hepatitis A. The household reported hosting a wedding at the home on 30 December 2018, where spring water was stored within the household before being served to guests. Six cases of hepatitis A were laboratory-confirmed among persons attending the wedding, not including the index cases among persons in the household who became ill 2 weeks before the wedding. The three unconfirmed illnesses were epidemiologically linked through spring water consumption or other exposure at the wedding. The eight remaining cases were identified among wedding attendees who went to hospitals in Tennessee (Allen County is in KY on the border of KY and TN) and were lost to follow up.

## MATERIALS AND METHODS

### Environmental investigation

An environmental investigation was conducted to evaluate the potential for spring water to contribute to the continued transmission of HAV in the community. This investigation consisted of site inspections, a sanitary survey, and drinking water and wastewater sample collection. ACHD initially visited the property in March of 2019 for sampling and the on-site environmental assessment, which included interviews with the property owner and assessment of the spring water location, flow, and patterns. Observations and inspection findings were documented on an environmental health outbreak investigation survey data collection tool. ACHD and the Kentucky Division of Water evaluated the resident’s black water tank used for all human fecal waste from the home for possible leaks in the system via a charcoal smoke test (National Association of Sewer Service Companies 2021). In December 2019, the property owners and downstream farms requested that ACHD retest the spring so they could commence using the spring water for irrigating produce crops sold locally. The ACHD conducted a subsequent site visit in January of 2020 for resampling water and wastewater to evaluate the persistence of viral shedding from the household and environmental contamination.

**Figure 2 |:**
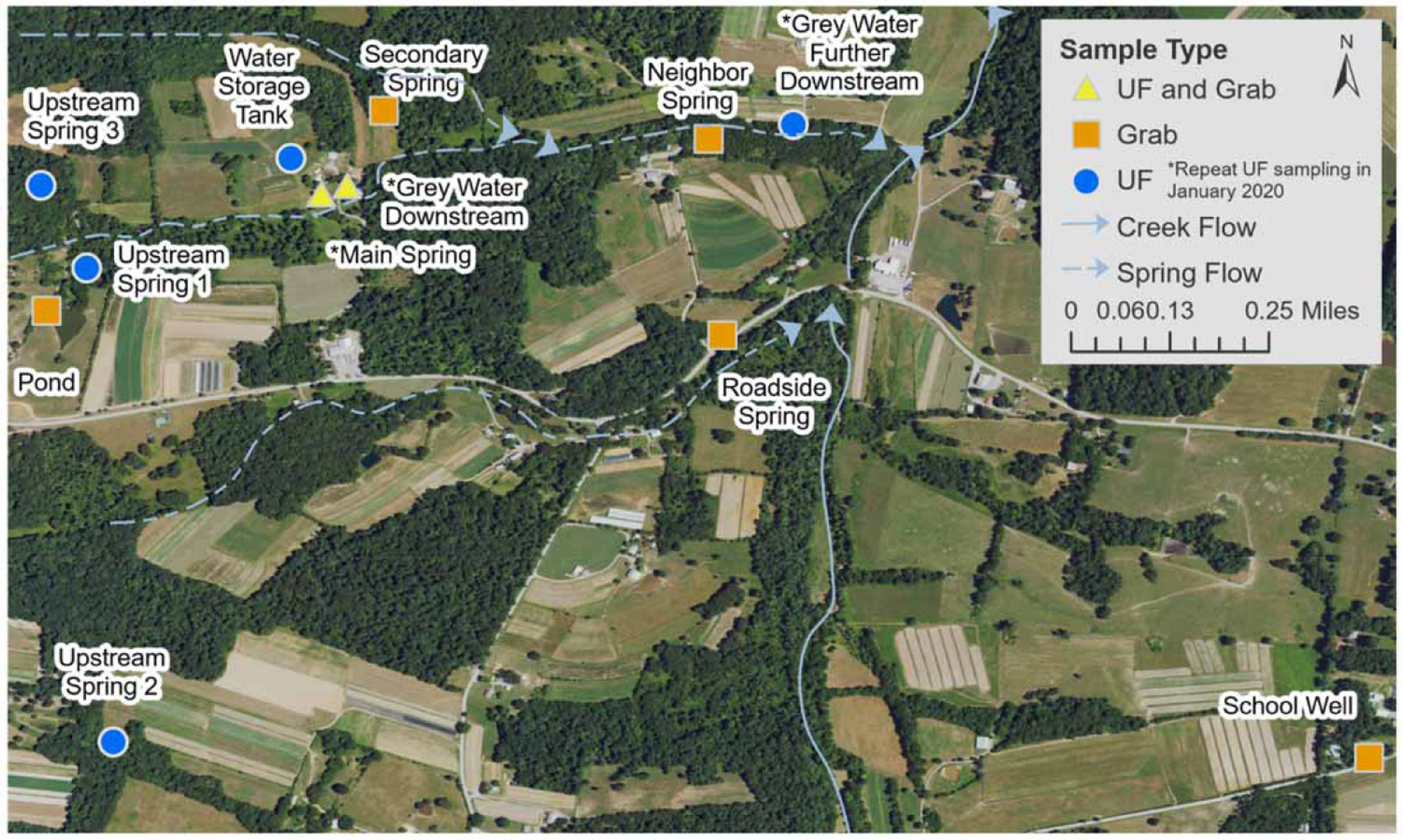
Map displaying sampling locations and spring water sites on the property and neighboring lots, Kentucky. UF, ultrafiltration water sample.

Environmental sampling: A map of the water sources and sampling sites is provided in Figure 2. On 11 March 2019, ACHD collected large volume (100-L) water samples through dead-end ultrafiltration (DEUF) (Gallardo *et al*. 2022) at two sites on the property for HAV testing (Jothikumar *et al*. 2005): (1) the site of drinking water collection from the main spring; and (2) immediately downstream of the home spring where the household gray water (i.e., non-human waste spent household water) discharged from a pipe (referred to as gray water downstream in Figure 2). The team also collected two small-volume (100-mL) water samples from each DEUF collection site for fecal indicator organism (i.e., total coliforms, *Escherichia coli (E. coli)*, and somatic coliphage) testing (Gallardo *et al*. 2022). A 100-mL grab sample was collected from a drinking well that served the community school for fecal indicator bacteria (FIB) (total coliforms and *E. coli* only) testing. The school groundwater well ~3 km (<2 mi) from the household where cases were identified was sampled due to concerns from the community that if the springs were contaminated there could be larger aquifer contamination; among outbreak cases, one case of illness was confirmed by laboratory testing for a schoolteacher and epidemiological links established for two students, one of which attended the wedding.

On 25 March 2019,ACHD collected five additional large volume DEUF samples for HAV testing from: (1) the three upstream spring feeding the home main spring; (2) the black water storage tank at the home; and (3) further downstream of the gray water discharge pipe. Four 100 mL grab samples were also collected from a nearby roadside spring, neighbor spring, secondary spring, and pond hydraulically feeding into the home main spring for somatic coliphage and FIB testing (Figure 2).

On 6 January 2020, ACHD conducted resampling for HAV using DEUF from three of the 2019 sampling sites: (1) the home main spring; (2) immediately downstream of the main spring and gray water discharge pipe; (Figure 2, referred to as gray water downstream); and (3) a site further downstream of the gray water discharge pipe near the neighbor’s spring. These sites were selected because the previously affected household and downstream farms wanted to resume spring water use for irrigating fresh produce crops. Paired small-volume (100-mL) water samples were also collected and tested for FIB and somatic coliphage, like the 2019 samples.

In late 2019, the black water tank was pumped out and filled with building materials to prevent reuse, and a new septic system was installed. On 17 February 2020, two wastewater samples (950 mL and 1 L) were collected from the household’s new septic tank for HAV and fecal indicator testing. Fecal indicator (generic and human-specific) testing of the septic tank was conducted to evaluate the abundance of these indicators within the small, on-site waste system to determine the validity in using these indicators for tracking human fecal waste from the system to nearby springs.

Water and wastewater samples were transported overnight to the CDC in Atlanta, GA, on ice for processing within 24 h of collection. At each sampling site, ACHD collected water temperature and pH using a Hach Multimeter Pocket Pro +, except for the school well and surrounding springs.

### Environmental sample processing and culture assays

In the initial 2019 environmental sampling, CDC tested DEUF water samples for HAV, total coliforms, *E. coli*, and the general viral fecal indicator, somatic coliphage. For the follow-up 2020 environmental testing, DEUF spring water and wastewater grab samples were tested for the 2019 microbes and also tested for the molecular-based human-specific viral fecal indicator, crAssphage (Stachler *et al*. 2017), to better track household human waste contamination to the spring. For all sampling events, total coliforms and *E. coli* were enumerated in 100-mL grab samples via the IDEXX QuantiTray-2000 method using Colilert-18 media (Standard Methods Committee 2018). Turbidity was also measured in grab samples from the four surrounding spring water samples using a Hach 2100P Turbidity Meter in nephelometric turbidity units (NTU).

DEUF samples were processed as previously described (Yard *et al*. 2014). Briefly, ultrafilters were first backflushed (~700 mL backflush concentrate volume). A portion of the DEUF backflush (20 mL) was tested directly for somatic coliphage using a modified version of US Environmental Protection Agency (USEPA) Method 1602 (U.S. Environmental Protection Agency 2001). The coliphage method was modified so that each plate contained 5 mL of sample in 30 mL of 1× media with the same concentrations of appropriate antibiotic, host, and MgCl_2_ in the final volume as Method 1602. Culture positive controls and media blanks were run in parallel to all samples. The following control strains were used for the culture-based microbial assays: 30 colony-forming unit (CFU) BioBalls^®^ (BTF Pty. Ltd, USA) for coliforms and *E. coli* (National Collection of Type Cultures 9001), and bacteriophage-ɸX174 (ATCC 13706-B1) for somatic coliphage (ATCC, Manassas, VA).

The remaining DEUF backflush was then subjected to secondary concentration by polyethylene glycol (PEG) precipitation and centrifugation (~3 mL final concentrate volume) as previously described (Yard *et al*. 2014). Briefly, PEG precipitation was conducted using 8% (wt./vol) PEG and 0.3 M (wt./vol) NaCl to the remaining backflush volume, incubated at room temperature for at least 2 h, and then repeatedly centrifuged in polycarbonate 250 mL bottles at 10,000 × g for 30 min at 4 °C. Supernatant was then removed and more sample added until all sample volume was concentrated. A portion of this concentrate (750 μL) was added to bead beating tubes and stored at 4 °C until nucleic acid extraction and testing for molecular targets (HAV and crAssphage). The remaining portion of the concentrate was also tested for somatic coliphage as described earlier, and volumes with countable plates were reported.

### Molecular assays

DEUF water sample concentrates (750 μL) were extracted for total nucleic acid as previously described with a final extraction elution volume of 80 μL for water samples and 50 μL for septic (Kahler *et al*. 2025). An extraction blank containing only reagents was run alongside the samples extracted from each sampling event. Extracts were stored for 24 h at 4 °C until molecular testing and stored at −80 °C until genomic analysis (if applicable).

HAV RNA was detected in water concentrates using reverse-transcriptase quantitative polymerase chain reaction (RT-qPCR) as described in Jothikumar *et al*. (2005). CrAssphage DNA (056 assay) was detected using qPCR as described in USEPA Method 1696 (U.S. Environmental Protection Agency 2019). Each sample was analyzed in triplicate with either 5 μL of extract in 50-μL reactions (RT-qPCR) or 2 μL of extract in 25-μL reactions (qPCR) and using either Taqman^™^ Fast Virus 1-Step Master Mix (ABI) (RT-qPCR) or Environmental MasterMix 2.0 [Applied Biosystems (ABI), Waltham, MA]) (qPCR), respectively, on an Applied Biosystems 7500 thermocycler (ABI). To test for amplification inhibition, each sample template was tested at a 1:10 dilution, and the presence of inhibition was defined by less than a 2.3 difference between *C*_q_ values for the diluted and undiluted samples (Boehm *et al*. 2013).

For each instrument run, the amplification threshold was set to 0.03 *Δ*Rn. Three no-template controls (NTC) were included with each instrument run to evaluate reagent contamination of the target. A (RT-)qPCR result was considered a detection if at least two reactions amplified at a quantification cycle (*C*_q_) value < 40 or if any amplified product was confirmed to be the target via sequencing. Otherwise, the result was considered a non-detect. CrAssphage efficiency and sensitivity were determined using standardized linearized synthetic DNA reference material (NIST SRM^®^ 2917) consisting of five 10-fold serial dilutions (10^1^ to 10^5^ copies per reaction) (Willis *et al*. 2022). CrAssphage concentrations were determined using the standard curve equation and volumes of sample processed and analyzed.

HAV detected in water samples underwent genomic analyses using targeted amplicon sequencing by the CDC Division of Viral Hepatitis laboratory. A 349-nucleotide segment of the HAV genome spanning the VP1–P2B junction was reverse transcribed, and the cDNA was PCR amplified and subjected to next-generation sequencing using the CDC-developed global hepatitis outbreak and surveillance technology (GHOST) protocol (Ramachandran *et al*. 2021). Genotyping and phylogenetic analysis using Neighbor joining algorithm and Kimura-80 Distance (CLC Genomics, Workbench v24, Qiagen, Aarhus) were conducted to determine the relatedness of the sequence to RNA-positive surveillance and outbreak samples collected in the US during the HAV outbreaks of 2016–2020 (Ramachandran *et al*. 2021; Foster *et al*. 2022).

## RESULTS

### Environmental assessment

The property where the wedding was held contained two homes; the second household was abandoned when the family moved away in December 2018, before the wedding. The spring and water collection site was located, 8 m (25 feet) down gradient from the primary home. The property residents utilized the main spring for household water use, including drinking. The spring water was reportedly transported and stored in coolers and tanks for home use. The spring water was also used to irrigate produce sold to the local community. After the initial outbreak during December 2018, the 22,000-L (7,000-gallon) drinking water storage tank was reportedly manually treated with chlorine in 2019.

The single outhouse used by the household where hepatitis A cases were identified was connected to a black water tank waste system. The black water storage tank was reportedly pumped out periodically by a separate company (frequency not reported). The charcoal smoke test did not detect leaks in the system. Gray water from the home was piped out into a stream created by the drinking water spring about 55 m (180 feet) downstream from the drinking water collection point. A large amount of heavy rainfall had been reported in the area during that winter.

### Environmental testing results

In March of 2019, HAV was detected by molecular-based methods (RT-qPCR) in both the main spring water and in the downstream water of the household gray water discharge pipe (both immediately and further downstream) (Table 1). The spring water had 1,203.3 most probable number (MPN) per 100 mL total coliforms and 58.3 MPN/100 mL *E. coli*. The water downstream of the household gray water pipe had 1,203.3 MPN/100 mL total coliforms and 102.2 MPN/100 mL *E. coli*. No FIBs were detected in the school groundwater well sample. HAV was also detected in the storage tank at the home. HAV was not detected in any of the three springs (Figure 2: Upstream Spring 1, 2, and 3) feeding the main spring, but somatic coliphage were detected in all three springs and the main spring (Upstream Spring 1, 2, and 3: 5.53, 2.21, and 5.2 plaque forming units/100 mL, respectively; main spring: 2.5 PFU/100 mL). Total coliforms, *E. coli*, and coliphage were also detected in the surrounding waters (Figure 2: roadside spring, neighbor spring, and secondary spring on the property).

**Figure 3 |:**
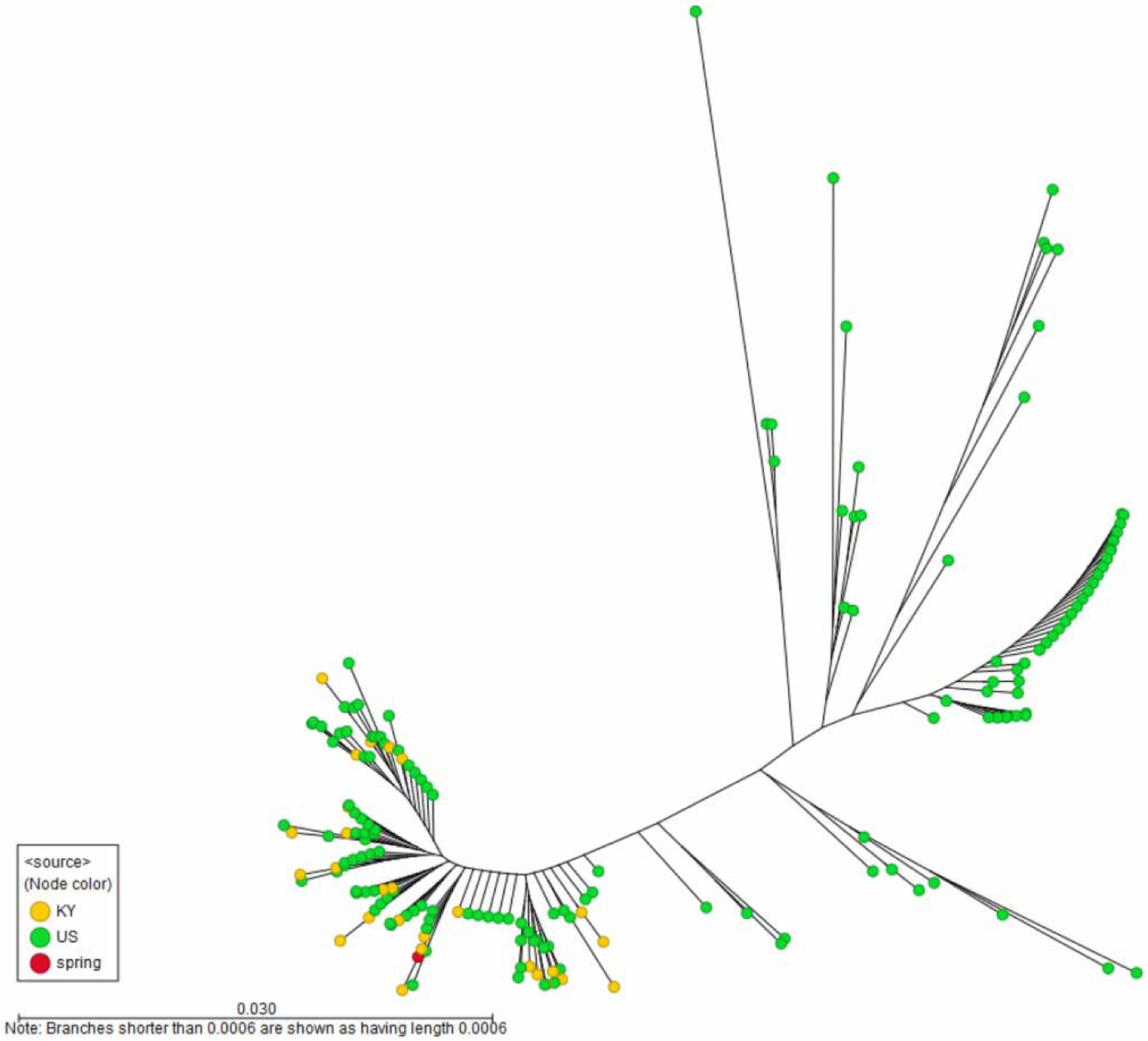
Phylogenetic analysis of the HAV strain detected in the spring water and its relatedness to unique clinical HAV sequences* from the USA in 2016–2020. *The leaves of the tree represent either single isolates or clusters (several cases with identical sequences); however, the frequency of each unique strain is not shown. Branch lengths that are 0, i.e., representing identical sequences, are given a value of 0.0006 (less than one nucleotide difference), that allows for better visualization, since identical sequences will otherwise overlap.

The sequence obtained from the spring water HAV detection covers a region spanning the VP1/P2B junction region of the HAV genome and identifies the detected HAV as genotype IB. The sequence was identical to a cluster of outbreak sequences obtained from Kentucky and very close to other unique genotype IB strains identified via surveillance or outbreak investigations in the United States throughout the period 2016–2020 (Ramachandran *et al*. 2021; Foster *et al*. 2022), as shown in Figure 3. The leaves of the tree represent either single isolates or clusters (several cases with identical sequences); however, the frequency of each unique strain is not shown.

From the 1-year follow-up testing in January of 2020, HAV was detected in the main spring and gray water downstream from the main spring (Table 2). Total coliforms and *E. coli* were detected in the main spring at levels above EPA drinking water standards (,1 *E. coli* per 100 mL) (2,419.6 and 58.5 MPN/100 mL, respectively). Somatic coliphage, viral fecal indicators, were also detected at all three sampling sites (main spring = 14.26 PFU/100 mL; downstream = 12.31 PFU/100 mL; neighbor spring = 25.45 PFU/100 mL), confirming viral-associated fecal contamination. The human-specific viral fecal indicator, crAssphage, was also detected in all three water samples; the concentration decreased farther downstream (Table 2).

In February of 2020, HAV was detected in the two small-volume wastewater samples collected from the newly installed, 3,800-L (1,000-gallon) septic tank (Table 2). Total coliforms and *E. coli* were at expected levels in the septic wastewater (total coliforms: 9.2–9.8 × 10^6^ MPN/100 mL; *E. coli*: 5.8–11.4 × 10^5^ MPN/100 mL), suggesting typical fecal input into the septic system. Somatic coliphage, viral fecal indicators, were also detected at expected levels in the septic wastewater (1.8–2.3 × 10^3^ PFU/100 mL). Finally, the human-specific viral fecal indicator, crAssphage, was detected in the septic wastewater samples (1.25 × 10^6^ copies/100 mL). Together, these data suggest that both the general and human-specific phages were indicators of septic wastewater from this household.

### Quality assurance/quality control

No contamination was observed in any culture-based indicator assays, nucleic extractions blanks, or NTC. Positive controls were detected as expected for all assays. (RT-)qPCR amplification inhibition was absent from all samples. The crAssphage pooled standard curve (*y* = −3.5347 *x* + 42.612) had an amplification efficiency *(E*) of 0.9183 (*E* = 10^(−1/slope−1)^) and *R*^2^ of 0.9967. The lower limit of quantification of the crAssphage assay had an average *C*_q_ of 38.95 (1 log10 copies per reaction).

## DISCUSSION

The environmental investigation confirmed wastewater contamination of the main spring used for household drinking where index cases of hepatitis A were identified. However, whether the household initial illness was exposed through contaminated water remains unknown since we do not know when the HAV entered the septic system and subsequently the spring water. While person-to-person transmission may have occurred at the wedding event, exposure to spring water may have also resulted in HAV infection for some household members. This is especially apparent given that the only household member who was not symptomatic and not diagnosed with hepatitis A was an adult man who did not drink spring water for medical reasons. Moreover, there were no additional cases among community members after the spring water was no longer used for household consumption or agricultural activities in March of 2019.

The long incubation period for hepatitis A (15–50 days) can be a barrier in investigating outbreaks (Nelson *et al*. 2020). Further, hepatitis A is self-limiting, and prolonged viral shedding is rarely reported, meaning a transient contamination event would be difficult to uncover after an outbreak (Castaneda *et al*. 2021; Migueres *et al*. 2021). However, this environmental investigation indicated a consistent contamination source of HAV into the drinking water at such a high concentration that it was detectable in the flowing spring, suggesting a hydrological connection between wastewater from infected individuals and the drinking water source. Prompt identification of the source of exposure is important in outbreak investigations because the hepatitis A vaccine should be administered within 2 weeks of exposure to all unvaccinated persons aged ≥12 months (Nelson *et al*. 2020), and mitigation is often required to prevent future cases.

HAV infection rates decreased nearly 97% during 1995–2015 after the introduction and widespread pediatric use of hepatitis A vaccines (Foster *et al*. 2022). However, since 2016, there has been an increase in HAV outbreaks in the United States (Foster *et al*. 2019; Castaneda *et al*. 2021; Foster *et al*. 2022). Illnesses associated with this increase occur predominantly among males, White persons, and those aged 30–49 years (Foster *et al*. 2022). The most frequently reported risk factor is drug use (Foster *et al*. 2022). In Kentucky, the KY DPH identified an outbreak of acute hepatitis A starting in November of 2017. Several of the cases were genetically linked to outbreaks in California, Utah, and Michigan (Kentucky Department for Public Health 2020; Hofmeister *et al*. 2021). Like the hepatitis A outbreaks in other states, the primary risk factors were recreational drug use and homelessness (Kentucky Department for Public Health 2020). While HAV RNA from the spring water-associated outbreak was closely genetically related to the nationwide outbreak sequence, the Kentucky cases did not have risk factors linked with the larger cluster (i.e., intravenous drug use or homelessness). This investigation highlights another risk factor group – unvaccinated persons who use water from untreated private springs that may be contaminated with human fecal matter (Barrett *et al*. 2019).

HAV has been detected in water and other environmental samples in previous outbreaks (De Serres *et al*. 1999; Tallon *et al*. 2008), but drinking water-associated outbreaks decreased sharply in the US since public health efforts like vaccination and drinking water regulations (Barrett *et al*. 2019; Nelson *et al*. 2020). Disinfection by chlorination or boiling is an effective way to inactivate HAV particles in water before use; otherwise, HAV can remain infectious for several weeks in water and sewage and even survive freezing (Cook *et al*. 2018). Anabaptist communities do not use modern sewage systems and retrieve water from non-treated non-municipal sources (Amraotkar *et al*. 2015). Consequently, studies have shown that communities similar to the Anabaptist community in Allen County, KY, were more likely to have detectable levels of *E. coli* and total coliforms in their drinking water than other rural communities using spring water sources (Amraotkar *et al*. 2015; U.S. Environmental Protection Agency 2024).

In this investigation, somatic coliphage and crAssphage results suggest subsurface septic intrusion (Murphy *et al*. 2020), resulting in virus-associated fecal contamination of the groundwater and connected spring water used for drinking (U.S. Environmental Protection Agency 2024). Many rural communities employ septic systems for wastewater disposal (Schaider *et al*. 2016), and these systems have been common contributing factors to outbreaks associated with untreated groundwater in the United States (Wallender *et al*. 2014). Home septic systems are not regulated by the US EPA (U.S. Environmental Protection Agency 2025) but instead are typically regulated by states. However, state-based regulations still do not often account for site-specific conditions, like land use, unique geological formations, and directional groundwater flow (Wilcox *et al*. 2010). Under Kentucky state law, the local health department issues permits to install septic systems (Kentucky General Assembly 2025). This outbreak property’s septic unit met the state standards of being at least 7.6 m (25 feet) from springs and streams (Kentucky General Assembly 2025). Still, HAV remained detectable in the spring water about 55 m (180 feet) downstream from the site of drinking water collection, indicating a hydrogeological connection between the spring water and black water tank, as well as the new septic system. This type of subsurface water contamination has been documented in other HAV outbreaks, such as one where HAV traveled 60 m (197 feet) from a cesspool to neighboring wells (De Serres *et al*. 1999) and another associated with a spring that was located downhill (distance unknown) from a farm’s septic tank (Tallon *et al*. 2008).

HAV was not detected in the upstream spring waters that feed into the main spring, further supporting the hypothesis that the source of contamination to the spring was on-site sanitation. The 2019 charcoal smoke study did not detect leaks in the black water tank waste system, but surrounding the outbreak period, there were two unique events that may have contributed to system failure. First, there was an 80% increase in the mean precipitation in the area during November 2018 (National Weather Service 2025). Heavy precipitation has been implicated in a previous spring water-associated HAV outbreak (Bergeisen *et al*. 1985). Second, the homeowners hosted a wedding on the property in December 2018. The heavy rainfall, combined with the addition of increased demand on the waste system, may have resulted in black water tank overflow. After the environmental investigation, the property owners began purchasing their water from municipal sources since HAV can persist weeks to months in water (Tallon *et al*. 2008; Cook *et al*. 2018), and continued contamination was a possibility. In late November/early December 2019, the black water tank was reportedly used past capacity again, then discontinued and filled with building materials to prevent further use. Subsequently, a new septic system was installed in late December 2019 before resampling; however, at the time of sampling, it was discovered that the lateral line to the system was installed too close to the spring and too deep, requiring repair, which may have contributed to the continued spring water HAV contamination.

In January and February 2020, HAV was again detected in the main spring water, as was the human-specific viral fecal indicator, crAssphage, indicating continued human fecal contamination of the spring. HAV can remain infectious on fresh vegetables for several days and for several months on frozen berries (Cook *et al*. 2018; Migueres *et al*. 2021). As recently as 2019 in the United States, there was a multi-state outbreak of HAV infections linked to fresh blackberries (McClure *et al*. 2022). Depending on temperature and humidity, HAV can remain infectious on surfaces for months and is known to be resistant to mild pasteurization (Cook *et al*. 2018; Migueres *et al*. 2021). Based on environmental testing, it was recommended that clinical specimens from all household members be collected to determine if there was either an asymptomatic shedder(s) or a symptomatic individual not receiving care remaining in the household. The family agreed to submit stool samples and complete a short questionnaire; however, by March of 2020, COVID-19 restrictions and resource limitations prevented follow-up to collect these specimens and administer a questionnaire.

## CONCLUSIONS

The environmental investigation and epidemiologic analysis of the risk factors associated with this hepatitis A outbreak provide evidence that the contaminated drinking water spring was an exposure source in this outbreak. Although we were unable to prove causality of exposure and infection through water individually in this outbreak, environmental testing and a sanitary survey determined that the spring water contamination likely originated from the waste system of the household where cases were identified, located directly uphill from the spring. The environmental detection of HAV and fecal indicators in the waste system, spring water, and downstream waters provided useful and timely information to assist with outbreak prevention and mitigation measures. In the event of a known biological contamination in drinking water, CDC recommends boiling for 1 min before use (Centers for Disease Control & Prevention 2024).

## Figures and Tables

**Table 1 |:** Environmental sampling results include physical and chemical water quality from the 2019 site visit, Kentucky

Sample location	Water quality parameters	Total coliforms (MPN/100 mL)	*E. coli* (MPN/100 mL)	Somatic coliphage (PFU/100 mL)	Hepatitis A virus (Detect/Non-detect)
Main Spring (UF and Grab)	Temp = 13.6 °C Turbidity = NA pH = 7.55	1,203.3	58.3	2.5	Detect
Gray Water Downstream of Main Spring (UF and Grab)	Temp = 13.6 °C Turbidity = NA pH = 7.55	1,203.3	102.2	<0.002	Detect
School Well (Grab)	NA	<1	<1	NA	NA
Gray Water Further Downstream of Main Spring (UF and Grab)	Temp = 13.8 °C Turbidity = NA pH = 8.13	NA	NA	39.5	Detect
Water Storage Tank (UF and Grab)	Temp = 17.5 °C Turbidity = NA pH = 7.89	NA	NA	0.39	Detect
Upstream Spring 1 (UF and Grab)	Temp = 17.2 °C Turbidity = NA pH = 7.49	NA	NA	5.53	Non-detect
Upstream Spring 2 (UF and Grab)	Temp = 16.0 °C Turbidity = NA pH = 7.28	NA	NA	2.21	Non-detect
Upstream Spring 3 (UF and Grab)	Temp = 15.1 °C Turbidity = NA pH = 6.91	NA	NA	5.2	Non-detect
Roadside Spring (Grab)	Temp = NA Turbidity = 7.91 NTU pH = NA	1413.6	133.4	6	NA
Neighbor Spring (Grab)	Temp = NA Turbidity = 3.08 NTU pH = NA	43.2	12.2	5	NA
Secondary Spring (Grab)	Temp = NA Turbidity = 2.85 NTU pH = NA	517.2	44.1	18	NA
Pond (Grab)	Temp = NA Turbidity = 20.5 NTU pH = NA	> 2,419.6	<1	17	NA

MPN: Most probable number, PFU: Plaque forming units, UF: Ultrafiltration water sample, NTU: Nephelometric units, NA: Not applicable/Data not available.

Ultrafilter samples represent ~100 L of water (Mull & Hill 2012).

Total coliforms and *E. coli* measured using EPA standard method, IDEXX Colilert-18, on 100 ml grab samples (Standard Methods Committee 2018).

Somatic coliphage measured using EPA Method 1602: Male-specific (F+) and Somatic Coliphage in Water by Single Agar Layer on 10 ml of ultrafilter backflush before secondary concentration (U.S. Environmental Protection Agency 2001). Further evaluated using molecular-based method, quantitative polymerase chain reaction (qPCR) (Stachler *et al*. 2017) to evaluate whether some portion of the culturable coliphage detected was human fecal in origin as crAssphage are human-specific coliphage.

Hepatitis A detected using molecular-based method, real-time reverse transcription quantitative polymerase chain reaction (Real-Time RT-qPCR) (Jothikumar *et al*. 2005).

**Table 2 |:** Environmental sampling results including physical and chemical water quality from the 2020 site visit, Kentucky

Sample location	Water quality parameters	Total coliforms (MPN/100 mL)	*E. coli* (MPN/100 m	Somatic coliphage (PFU/100 mL)	crAssphage Copies/100 mL)	Hepatitis A virus (detect/non-detect)
Main Spring (UF and Grab)	Temp = 15.4 °C Turbidity = 4.17 NTU pH = 7.6	2,419.6	58.5	14.26	1.25	Detect
Gray Water Downstream of Main Spring(UF and Grab)	Temp = 16.1 °C Turbidity = 6.47 NTU pH = 7.6	2,419.6	156.5	12.31	0.34	Detect
Gray Water Further Downstream of Main Spring (UF and Grab)	Temp = 12.6 °C Turbidity = 4.27 NTU pH = 8.02	2,419.6	204.6	25.45	Non-detect	Non-detect
Septic Tank Sample 1	Temperature NA Turbidity = NA pH NA	9,208,000	1,137,000	2,300	1.25 × 10^6^	Detect
Septic Tank Sample 2	Temperature NA Turbidity = NA pH = NA	9,804,000	576,000	1,800	1.25 × 10^6^	Detect

MPN, most probable number; PFU, plaque-forming units; UF, ultrafiltration water sample; NTU, nephelometric units; NA, not applicable/data not available.

Ultrafilter samples represent ~100 L of water (Mull & Hill 2012).

Total coliforms and *E. coli* were measured using the EPA standard method, IDEXX Colilert-18, on 100 mL grab samples (Standard Methods Committee 2018).

Somatic coliphage measured using EPA Method 1602: Male-specific (F+) and Somatic Coliphage in Water by Single Agar Layer on 10 ml of ultrafilter backflush before secondary concentration (U.S. Environmental Protection Agency 2001).

crAssphage measured using molecular-based method, quantitative polymerase chain reaction (qPCR) (Stachler *et al*. 2017).

Hepatitis A detected using molecular-based method, real-time reverse transcription quantitative polymerase chain reaction (Real-Time RT-qPCR) (Jothikumar *et al*. 2005).

## Data Availability

All relevant data are included in the paper or its Supplementary Information.

## HIGHLIGHTS

- Reported drinking water-associated hepatitis A outbreaks are uncommon, but unvaccinated persons who use water from untreated sources remain at risk.
- Private springs are unregulated in the United States and should be considered a risk factor in HAV infection.
- Public health officials could raise awareness of risks associated with untreated ground water among users and provide options for water testing and treatment.
